# Toe-tourniquet syndrome: A rare potentially devastating entity

**DOI:** 10.5704/MOJ.1511.008

**Published:** 2015-11

**Authors:** N Baloch, M Atif, RH Rashid, PM Hashmi

**Affiliations:** Department of Orthopaedic Surgery, Aga Khan University Hospital, Karachi, Pakistan

**Keywords:** Toe, Tourniquet, Child, Hair, Gangrene

## Abstract

Toe-tourniquet syndrome is a rare and commonly misdiagnosed condition caused by a hair or a fiber wrapped around digits (fingers and toes). A four months baby girl who was crying and presented with redness and swelling at her 2nd and 3rd toes of right foot. Child had red and swollen 2nd and 3rd toes of right foot with hair end protruding through wounds. Constricting hairs were cut and removed. Toetourniquet syndrome is a rare entity which is caused by hair wrapped around a toe or a digit. Diagnosis is mostly clinical. In order to prevent this condition to happen, education of parents and clinicians is a cornerstone.

## Introduction

Toe-tourniquet syndrome, also called Hair-thread tourniquet syndrome (HTTS), is a rare and commonly misdiagnosed condition caused by hair or fiberwrapped around digits (fingers and toes), penis, or even clitoris. It usually affects infant and children.

### Treatment:

It includes early recognition of condition (clinical diagnosis) and immediate release of constriction to prevent devastating complication in the form of toe or finger loss (amputation). Careful circumferential examination of affected part should be done as swelling and erythema, can mimic infection leading to devastating results. In doubtful cases hand held magnifying glass can be useful.

A longitudinal (peritendinous) incision can be given. As children have low immunity, antibiotic can be given safely. Usually wound heals uneventfully but some form of flexion deformity can be seen as complication.

## Case Report

**Fig. 1:**
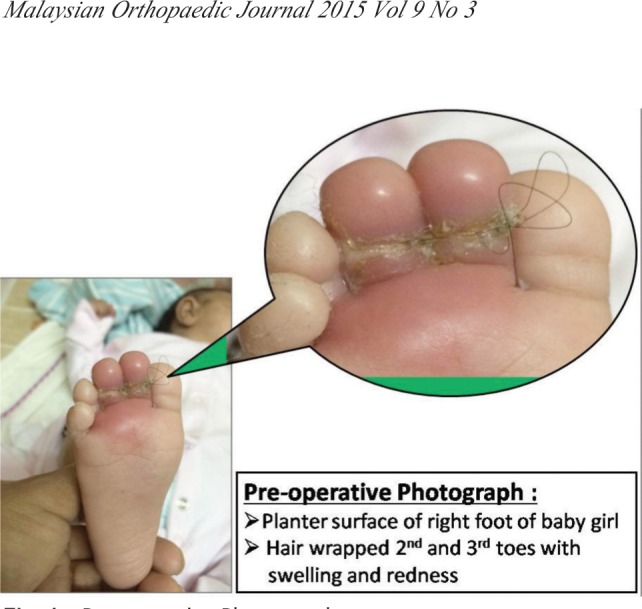
Pre-operative Photographs.

A four months baby girl, presented in emergency, having redness and swelling at her 2nd and 3rd toes of right foot. Mother said that child was irritable a day before and crying with no obvious reason. On examination child had a deep circumferential groove over 2nd and 3rd toes of right foot, toes were red and swollen with hair end protruding through the wounds (Figure 1a). There was no recent infection, no known congenital malformation (condition), fever or trauma. Hair was wrapping the toe digits in figure of eight position bringing the toes together. Constricting parts of hair were embedded deep in the wound. After counseling of the family, patient was shifted to Operation Theater. Under general anesthesia, using loupes (3x magnification), hairs were removed (Figure 1b). Wound at lateral surface of 2nd toe and medial surface of 3rd toe was only skin deep and rest of surface was bone deep. That can be explained by figure of eight configuration of hair wrapping both toes and that may have saved digital neurovascular on lateral side of 2nd toe and medial side of 3rd toe. Capillary refill was found to be normal in both toes.

Postoperatively the toes were well perfused and the patient was discharged on 1stpost-operative day (Figure 1c). Antibiotic was given for 5 days.

## Discussion

Wrapping of hair or fiber produces tourniquet syndrome like situation which causes reduction in venous and lymphatic drainage causing swelling, edema and congestion of affected part of body. This ultimately results in pressure on arteries (end arteries in case of toes and fingers) producing ischemia and gangrene with end result of amputation.

Although Toe-tourniquet syndrome is a rare entity (one center reported the annual incidence as 0.02%)^3^ but a clinician should be familiars with it. Diagnosis is clinical but can be misinterprets as infection, trauma, insect bite, allergic or contact dermatitis. Careful examination of digits for strangulation in an irritable child with digit swelling is a crucial step.

**Fig. 2:**
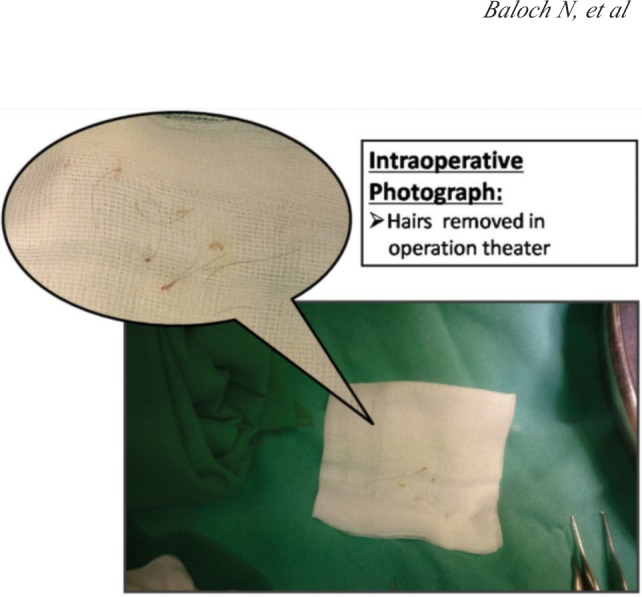
Intraoperative photograph.

**Fig. 3:**
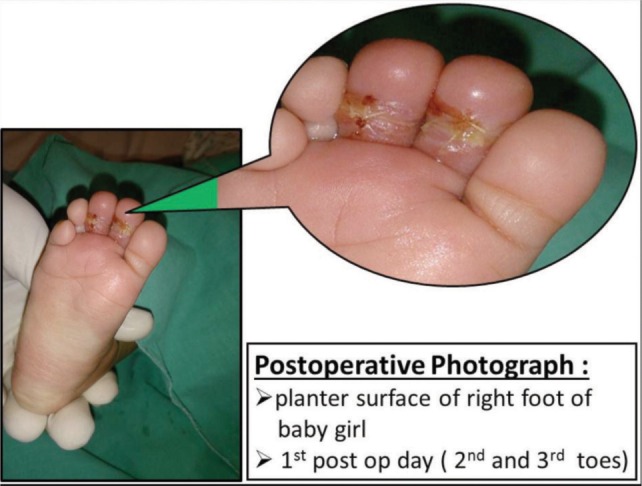
Postoperative Photographs.

It was Quinn who first described the term “Toe-tourniquet Syndrome” in 1971^1^. Other authors described it as Hair-thread tourniquet syndrome. It is a surgical emergency and causes ischemic strangulation of toe, a compartment syndrome like condition, failure of its recognition and early intervention leads to amputation of body part.

There are many risk factors associated with this condition. Literature showed that women going through pregnancy can experience sever hair loss^2^. After child birth hair growth become normal in about 4-6 months. This commonly occurs in 90% of postpartum women. It is called “Telogen Effulvium”. Being a rare entity it is very difficult to comment on whether it is neglect or an abuse. Neglect while giving bath and wrapping the child without checking appendages carefully should also be considered. Summer season is considered to be a one of the contributing risk factors. Babies are less covered with cloths especially fingers and toes during summer as compared to winter. Carpets are also considered as one of the risk factors

Goal of treatment is to remove constricting hair bands. Prompt removal of constriction even in emergency room is important. In case of failure, patient must be made to move to Operation Theater. Different techniques have been described like O’ Gorman and Ratnapalan^4^ have used a depilatory cream for hair removal concluding that it is very effective and a safe method, but is applicable for only superficial hairs and not for the deeper ones.

Serour and Gorenstein^5^ described a surgical technique in which a peritendinous incision is given, over the area of strangulation on dorsal aspect of toe as for as proximal phalanx bone. Complete transection and release of constricting hair fibers is recommended. In our case, we removed hair with surgical blade using magnifying Loup’s (3x) and with help of microsurgery instruments.

## Conclusion

Toe-tourniquet syndrome is a rare entity and the diagnosis is clinical. It should be one of the differential diagnosis of acute swollen toes in young child. Evidence of tip of hair is a characteristic feature. Early proper treatment leads to good outcome.

## Consent

Written informed consent was obtained from parents for publication of this case report and any accompanying images.
